# Subretinal Worm and Repeat Laser Photocoagulation

**DOI:** 10.4103/0974-9233.63072

**Published:** 2010

**Authors:** Sribhargava Natesh, Harsha K, Unnikrishna Nair, KGR Nair

**Affiliations:** Narayana Nethralaya Super Speciality Eye Hospital, Post Graduate Institute of Ophthalmology, 121/C, Chord Road, Rajajinagar, Bangalore-560010, India; 1 Chaithanya Eye Hospital, Kesavadasapuram, Trivendrum, Kerala, India

**Keywords:** Diffuse Unilateral Subacute Neuroretinitis, Sub Retinal Worm, Repeat Laser Photocoagulation

## Abstract

Diffuse unilateral subacute neuroretinitis (DUSN) can be a diagnostic dilemma. Laser photocoagulation of the subretinal worm is an effective treatment for eradication. Early laser photocoagulation has been advocated. We report a case of a middle aged man who presented with decreased vision and a sub retinal macular worm that required two laser sessions for complete eradication of the worm.

## INTRODUCTION

Diffuse unilateral subacute neuroretinitis (DUSN) is a parasitic disease affecting the outer retina and retinal pigment epithelium (RPE) that occurs unilaterally or bilaterally.^1^ Macular cysts have been reported due to the subretinal worm.^2^ Parasites of different sizes and several species of nematodes have been reported as etiologic agents of DUSN including *Toxocara canis*, *Ancylostoma caninum*, *Strongyloides stercoralis, Ascaris lumbricoides* and *Baylisascaris procyonis*. It is established that DUSN can be caused by two different sized nematodes, the smaller measuring 500 μm and the larger measuring between 1500 μm and 2000 μm.^3^ Gass suspected that the smaller worm was *Ancylostoma caninum*, a canine parasite that causes cutaneous larva migrans in humans.^4^ Kazacos and colleagues identified the larger worm as being *Baylisascaris procyonis*, found in the intestine of raccoons and skunks.^5^,^6^ However, the clinical sign and symptoms are the same for both parastic infections.

Presenting symptoms are visual blurring and loss, vitritis, disc edema and RPE tracks including white to grey lesions. Patients lose vision due to macular RPE changes and optic atrophy with arteriolar narrowing. The definitive treatment modalities include oral anthelminthics, (albendazole 400 mg/day for 30 consecutive days) oral corticosteroids and early laser photocoagulation.^7^–^9^ However, there are no reports on laser retreatment in the peer reviewed literature. In this case report, we present a rare case of DUSN where laser retreatment of the subretinal worm resulted in obliteration of the worm.

## CASE REPORT

**Figure 1:**
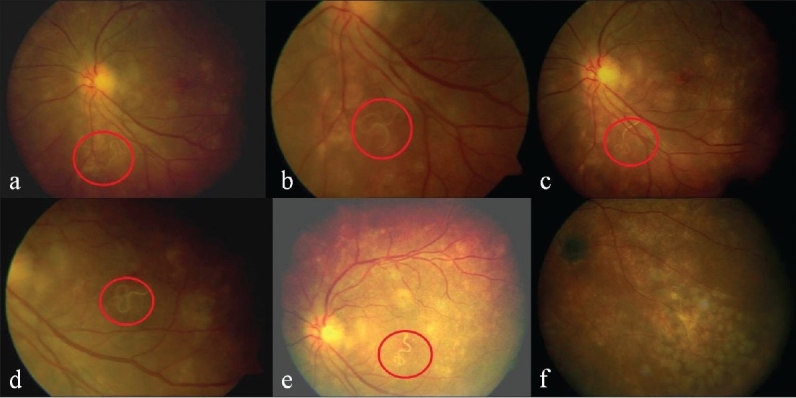
(a-e) Migration of the subretinal worm across the fovea; (f) Dead worm after laser treatment in the inferotemporal quadrant after partially moving from the original retinal area of laser treatment. Note the black granuloma

A middle-aged healthy man from southern India presented with complaints of visual blurring and floaters for one month. He was an agriculturist who worked bare foot in the fields and denied history of foreign travel. The right eye examination was within normal limits. The vision was 20/200 in the left eye with a relative afferent pupillary defect and vitreous cells. The left eye had disc edema with blurring of the margins, RPE grey-white lesions in the mid periphery and the macular area and tracks. A black granuloma the size of one disk diameter was noted along the inferotemporal arcade that was likely the site of larva. A motile subretinal worm was located in the inferior retina [Figure 1a–e]. During fundus photography, the worm moved across the fovea due to light stimulation. After 6–7 minutes worm moved out of the macular area, to the inferotemporal quadrant. It was photocoagulated with a neodymium-doped yttrium aluminium garnet (Nd:YAG) double frequency 532 nm green laser delivering a few burns on the worm. Laser barrage (260 mw power, 100ms duration and 208 burns using an indirect laser ophthalmoscope) was then performed to prevent migration of the worm and as it was highly motile. The worm was immobilized after photocoagulation and the patient was treated with oral anthelminthic agents (single dose of Tablet albendazole 400 mg) to treat any intestinal infestation and a short course of oral prednisolone (Wysolone, Wyeth Laboratories) over 2 weeks (50 mg of initial dose that was tapered by 20 mg every 5 days).

Patient was seen the following day and despite treatment the worm had moved out of the lasered area and one end of the worm was motile [Figure 1f]. Additional laser burns were delivered to kill the worm and subsequently patient's vision improved to 20/40 over 15 days.

## DISCUSSION

We present an interesting case of a subretinal worm that migrated in the subretinal space. The worm in our case can be Baylisascaris or Ascaris due to the size and the propensity to migrate. However, it was unlikely that the worm was Baylisascari procyonis since its typical hosts, skunks and raccoons, are not found in southern India.^3^

Laser photocoagulation is an effective method to treat subretinal worms and early treatment has been shown to halt the progression of the worm and improve visual acuity.^9^ In areas of RPE atrophy and retinal edema as in this case, it may be difficult to recognise whether the laser burns were adequate to kill the worm and in such cases, it is prudent to examine the patient and repeat laser treatment if needed. The over treatment with laser, evidenced from the examination after initial treatment, was due to the fact that the worm was highly motile and the anxiety of the surgeon not to miss the worm. It is typically few laser burns that are required to treat the worm. It is however prudent to deliver laser treatment to the worm once it migrates away from macula and in the inferior quadrant to prevent a scotoma in the central and the inferior field.

Longer duration treatment with albendazole has been described in retinal involvement with worms akin to the treatment of neurocysticercosis without laser treatment.^7^ The role of oral anthelminthics in conjunction with lasers is not clear. We treated the patient with single dose of oral albendazole to eliminate any intestinal tract infestation.

There are reports of progressive optic atrophy with DUSN.^7^ Our patient had a relative afferent pupillary defect, vitritis and papillitis and hence we treated the patient with a two week tapering doses of oral corticosteroids. In some cases, the use of oral anthelminthics and corticosteroids may reduce the media haze and enable the recognition of small worms. In our patient, use of oral corticosteroids may have reduced the inflammation and papillitis facilitating rapid visual recovery.

We suggest that laser treatment for the subretinal worm is effective and recommend careful repeat examination in all cases to ensure that the worm is immobile and repeat laser treatment when warranted.
